# Surviving the Test of Time: A Young Patient’s Triumph Over Early-Onset Invasive Ductal Carcinoma and Its Recurrence a Decade Later

**DOI:** 10.7759/cureus.42613

**Published:** 2023-07-28

**Authors:** Muhammad Hamza Shah, Mushahida Batool

**Affiliations:** 1 Medical School, Dentistry & Biomedical Sciences, Queen's University Belfast, Belfast, GBR; 2 General & Breast Surgery, Omar Hospital & Cardiac Centre, Lahore, PAK

**Keywords:** recurrence, genetic predisposition, modified radical mastectomy, cancer survivorship, ductal carcinoma

## Abstract

Breast cancer is a complex, heterogeneous disease with diverse clinical presentations and variable outcomes. In this report, we provide a detailed analysis of a case involving a 22-year-old woman diagnosed with invasive ductal carcinoma, highlighting the difficulties of managing breast cancer in young patients. Through the examination of this patient's 10-year journey, from the initial diagnosis to surgery, adjuvant therapy, and recurrence, we underline the crucial role of early detection, personalised treatment, and interdisciplinary cooperation in optimising patient outcomes. Overall, the case study serves as a compelling narrative, effectively highlighting the aggressive nature of breast cancer in younger individuals and underscoring the need to provide care that addresses the multifaceted dimensions of this disease.

## Introduction

This report aims to provide insights into the clinical course, management strategies, and outcomes in an atypical breast cancer scenario with a young patient. According to the World Health Organization (WHO), breast cancer has now surpassed lung cancer as the world’s most commonly diagnosed cancer, with nearly 2.3 million new cases in 2020 [1]. Incidence and mortality rates of breast cancer exhibit substantial variability across different regions, with the highest incidence rates reported in Western Europe and North America and the lowest rates observed in Eastern and Middle Africa [2]. In contrast, mortality rates demonstrate an inverse relationship, with higher rates observed in low and middle-income countries (LMICs) compared to developed nations.

Breast cancer, despite the common perception that it is a disease of postmenopausal women, is not exclusive to any age group. Indeed, it constitutes a noteworthy clinical issue among young women, underlining the significance of clinical vigilance and prompt diagnostic assessment. In Pakistan, a staggering one in nine women faces the risk of being diagnosed with breast cancer at some point in their lives [3]. Even more alarming is that over 25% of these cases occur in women under the age of 40 at the time of diagnosis [4]. Furthermore, breast cancer in young women can present unique challenges, including considerations related to fertility, family planning, genetic predisposition, and long-term survivorship, which adds layers of complexity to its management and underscores the need for comprehensive care. Ductal carcinoma, specifically, is the most common subtype of breast cancer, representing approximately 50%-70% of all breast cancer diagnoses globally [5]. This type of carcinoma originates from the cells lining the milk ducts and can manifest as either in situ (confined within the ducts) or invasive (when abnormal cells in the milk ducts invade breast tissue beyond the duct walls).

This report discusses the case of a 22-year-old woman presenting with a progressively enlarging right breast lump, later confirmed as invasive ductal carcinoma. We discuss her clinical journey spanning a decade, from the initial diagnosis to surgical management, adjuvant therapy, and recurrence, aiming to highlight the complexities of managing breast cancer in young women and the critical role of interdisciplinary collaboration in ensuring optimal outcomes. The journey of survival and the multidisciplinary approach employed in this patient's care underline the importance of early detection, tailored treatment, and ongoing surveillance for breast cancer in young individuals.

## Case presentation

We report the case of a 22-year-old woman who sought medical attention for a gradually enlarging lump in her right breast that she had noticed approximately two months prior to presentation. The patient reported no pain associated with the lump or any nipple discharge. Her menstrual cycles were regular, and she was unmarried at the time of the presentation. Her medical history was unremarkable, void of any comorbidities or past surgical interventions. A salient feature in her family history was the significant prevalence of breast cancer, involving three first-degree relatives (two paternal aunts and one first cousin) and extending to two second-degree cousins, suggesting a potential genetic predisposition. Due to the lack of educational awareness regarding screening and the absence of a standardised screening programme in Pakistan, the patient didn't undergo mammography screening despite her extensive family history.

Upon clinical assessment, the patient presented with overall stable health. A detailed examination of the breast region revealed a palpable, hard, irregular mass measuring approximately 5.7 x 5 cm. The small size of the breast accentuated the mass, which was noted to be retroareolar in location, displaying no signs of adherence to the overlying cutaneous layer or to underlying structures. Visual inspection illuminated an apparent fullness on the right side of the chest. Furthermore, upon axillary evaluation, palpable lymph nodes were identified on the right axilla. Her systemic examination, encompassing abdominal, respiratory, cardiovascular, and central nervous system components, remained within normal limits.

In order to evaluate the potential extent of the disease, a metastatic workup was initiated. Baseline blood investigations were all within normal parameters, and biomarkers weren't ordered due to the lack of availability and high cost associated with acquiring them through private means. A chest X-ray and a comprehensive CT chest, abdomen, and pelvis (CAP) for staging all yielded negative results. Additionally, a bone isotope scan exhibited no evidence of skeletal metastases. Cardiac function was evaluated by echocardiography, with normal findings reported. Following the comprehensive investigations, the case was presented to the oncology team and pathologists for interdisciplinary deliberations. Table 1 summarises the pathology report and includes results from the fine needle aspiration cytology (FNAC).

**Table 1:** The patient's pathological findings

Specimen	Fine needle aspirate from the lump in the right breast for cytology X 2 (samples)
Microscopy	Smears from these aspirates contain blood, macrophages, and many degenerate cells (++). In addition, there are many single cells, groups, and sheets of pleomorphic malignant epithelial cells, revealing a high nuclear/cytoplasmic (N/C) ratio, and hyperchromatic nuclei with nucleoli.
Histopathology	Histologic grade: Grade III primary tumour: PT2 (>2.0cm) regional lymph nodes: Pn1a (metastasis in 1 to 3 axillary lymph nodes – 3/15). Margins: Margins uninvolved by invasive carcinoma distance from the closest margin: 2.0mm
Immunochemical Status	Estrogen receptor (ER): negative; Progesterone receptor (PR): negative; Human epidermal growth factor receptor 2 (HER2): negative
Diagnosis	Aspirate from cancer (CA) breast (most likely ductal in origin)

Based on the findings and discussion with the patient and her family, the decision was made for surgical intervention in the form of a unilateral Modified Radical Mastectomy (MRM). Given the retroareolar location of the tumour, an elliptical incision was made, ensuring adequate safety margins. Flaps were raised to the anatomical borders of the clavicle and the 8th rib inferiorly, the midsternal line medially, and the mid-axillary line laterally. Level 3 axillary dissection was performed, following which hemostasis was ensured and a single drain was placed in situ.

**Figure 1:**
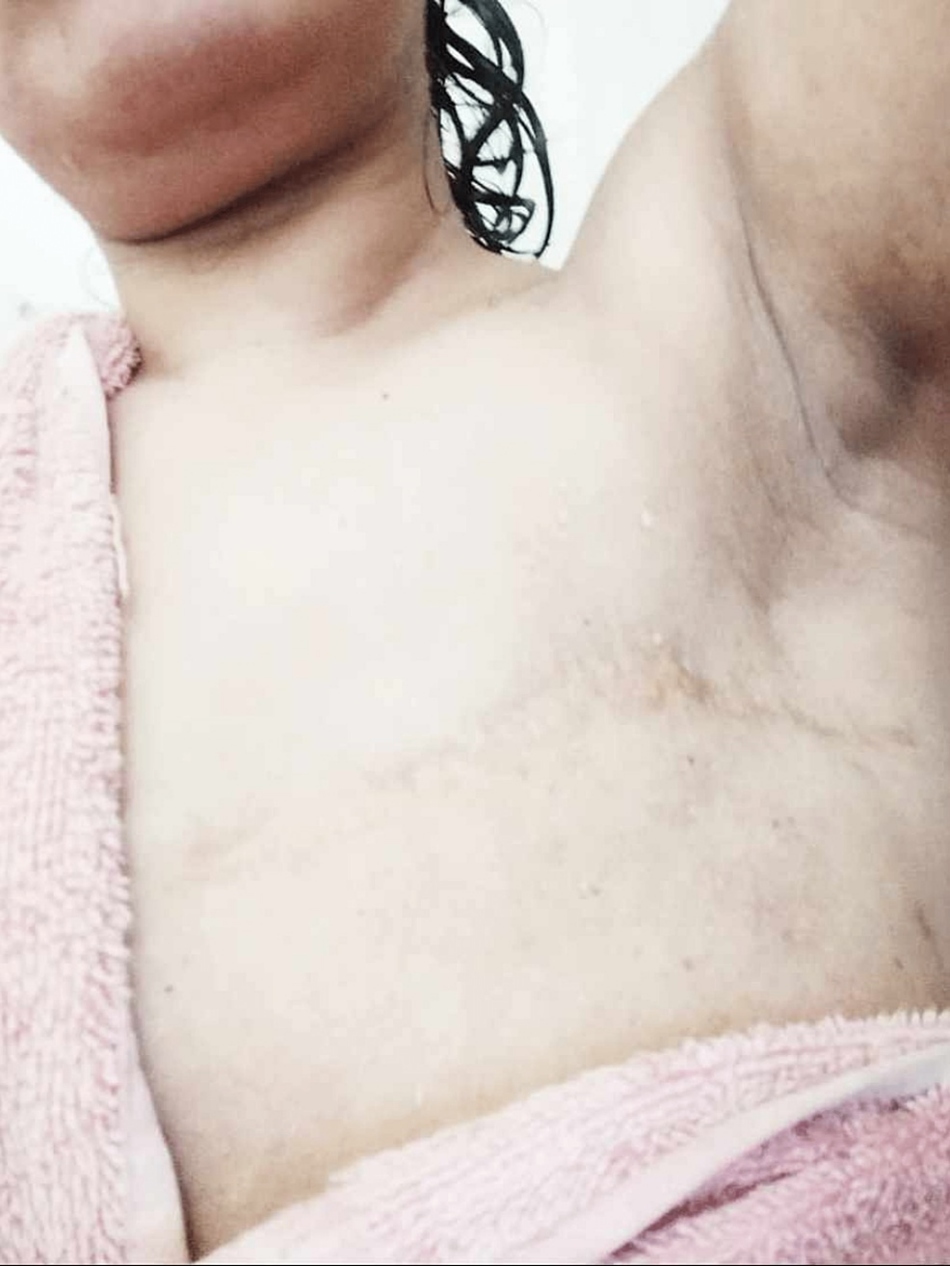
Healed post-mastectomy incision site

Subsequent to the surgical procedure, the patient was managed with adjuvant chemotherapy, comprising six cycles of the paclitaxel-cyclophosphamide combination regimen, under the supervision of the oncology team. The patient was then placed under regular follow-up by both the surgeon and oncologist for a span of 10 years, during which her yearly follow-up mammography was within normal limits. During this 10-year period, the patient got married and, on the recommendation of her oncologist, decided to complete her family with one child. Initially, fertility preservation was recommended, but since the patient ultimately had three children in a relatively short span of time (all of them being normal deliveries and soon after her chemotherapy finished), this was not continued. Figure 1 reveals the operating site and incision on her latest visit to the clinic, 15 years after the initial Modified Radical Mastectomy (MRM).

Recurrence

Ten years following her initial presentation, she noticed a lump in the opposite breast during a self-examination. This lump, measuring 0.5 x 1 cm, was investigated thoroughly, and the triple assessment, including an ultrasound-guided core tissue biopsy, was repeated by the operating surgeon. The results revealed a recurrence of ductal carcinoma on the opposite side (likely to be another primary disease). After a thorough discussion of the treatment options and despite the recommendation for another mastectomy, the patient opted for breast conservation since she had been struggling with the psychosocial aspects of her prior mastectomy. Due to logistical considerations, this procedure was performed by a fellow surgeon at a different institution.

## Discussion

Breast cancer is a complex disease with varying clinical presentations and outcomes. In this case report, we present a recurrence of ductal carcinoma in a young patient who had previously undergone a mastectomy. The aggressive nature of breast cancer in younger patients is particularly notable, with worse prognostic indices, including poor cell differentiation and hormonal insensitivity, contributing to the challenges in managing this population [6]. Furthermore, studies have shown that a younger age at diagnosis is associated with increased tumour size and more frequent lymph node involvement [7]. These factors collectively contribute to a worse prognosis and may necessitate more aggressive treatment strategies.

Interestingly, lifestyle risk factors have been known to play a significant role in breast cancer development among young women. Physical activity has been associated with a dose-dependent reduction in the risk of breast cancer, contradicting earlier studies that suggested no association [8]. Increased BMI has also been found to have a modest protective effect against breast cancer in young women, although the risk reduction may be offset by an increased post-menopausal risk later in life [9]. Alcohol consumption has also been consistently associated with an increased risk of breast cancer in young women, with a dose-response effect observed [10]. Conversely, the impact of smoking on breast cancer risk remains unclear, with some evidence suggesting a potential association, particularly in premenopausal women. Passive smoking may pose a greater risk for breast cancer in young women compared to active smoking, but the evidence only shows a weak correlation [11]. Further research is needed to elucidate the precise role of these lifestyle factors and their interactions in breast cancer risk among young women.

Additionally, it’s important to note that breast cancer incidence and mortality rates among young women exhibit significant disparities between developed countries and low- and middle-income countries (LMICs) [12]. Differences in screening practices may contribute to the difference, as mammography screening is recommended at an earlier age in developed countries compared to LMICs. However, it's also worth noting that overdiagnosis is a concern in screening programmes, with a significant proportion of diagnosed breast cancers being potentially indolent and not clinically apparent in the absence of screening.

In light of the points above, further studies are warranted to enhance our understanding of breast cancer biology in younger patients and refine treatment approaches to improve outcomes in this high-risk population.

## Conclusions

This case study serves as a poignant reminder of the crucial role played by early detection, personalised treatment, and ongoing surveillance in effectively managing breast cancer in young patients. It underscores the importance of considering factors such as fertility preservation, family planning, genetic predisposition, and long-term survivorship when making treatment decisions. The key takeaway is that comprehensive care, encompassing medical, surgical, and psychosocial aspects, is vital for meeting the diverse needs of these patients and achieving optimal outcomes. By integrating these essential components into a comprehensive care framework, we can strive towards improved prognoses and a better quality of life for young breast cancer patients.
